# Community-Based Matrix Factorization (CBMF) Approach for Enhancing Quality of Recommendations

**DOI:** 10.3390/e25091360

**Published:** 2023-09-20

**Authors:** Srilatha Tokala, Murali Krishna Enduri, T. Jaya Lakshmi, Hemlata Sharma

**Affiliations:** 1 Algorithms and Complexity Theory Lab, Department of Computer Science and Engineering, SRM University-AP, Amaravati 522502, India; srilatha_tokala@srmap.edu.in (S.T.); muralikrishna.e@srmap.edu.in (M.K.E.); jayalakshmi.t@srmap.edu.in (T.J.L.); 2 Department of Computing, Sheffield Hallam University, Howard Street, Sheffield S1 1WB, UK

**Keywords:** matrix factorization, recommender system, community detection, rating network, RMSE

## Abstract

Matrix factorization is a long-established method employed for analyzing and extracting valuable insight recommendations from complex networks containing user ratings. The execution time and computational resources demanded by these algorithms pose limitations when confronted with large datasets. Community detection algorithms play a crucial role in identifying groups and communities within intricate networks. To overcome the challenge of extensive computing resources with matrix factorization techniques, we present a novel framework that utilizes the inherent community information of the rating network. Our proposed approach, named Community-Based Matrix Factorization (CBMF), has the following steps: (1) Model the rating network as a complex bipartite network. (2) Divide the network into communities. (3) Extract the rating matrices pertaining only to those communities and apply MF on these matrices in parallel. (4) Merge the predicted rating matrices belonging to communities and evaluate the root mean square error (RMSE). In our experimentation, we use basic MF, SVD++, and FANMF for matrix factorization, and the Louvain algorithm is used for community division. The experimental evaluation on six datasets shows that the proposed CBMF enhances the quality of recommendations in each case. In the MovieLens 100K dataset, RMSE has been reduced to 0.21 from 1.26 using SVD++ by dividing the network into 25 communities. A similar reduction in RMSE is observed for the datasets of FilmTrust, Jester, Wikilens, Good Books, and Cell Phone.

## 1. Introduction

To predict recommendations to the users based on past behavior and their preferences, a technique in recommender systems named collaborative filtering is used [1]. The main objective is to suggest items or content to the users by considering their interactions or resemblances with other users. Due to the continuous growth in data availability and the interconnected nature of diverse systems, these techniques hold immense importance in revealing patterns, relationships, and significant insights [2].

Matrix factorization (MF) is a technique utilized in collaborative filtering to decompose a matrix of user-item ratings into lower-rank matrices capturing the latent factors underlying the data [3,4]. The user-item rating matrix serves as a representation of user ratings assigned to different items [5]. The matrix is frequently sparse as users typically only rate a small subset of items. MF techniques strive to complete the missing entries in the matrix by decomposing it into lower-rank matrices, one capturing the user’s underlying preferences and the other reflecting the item’s latent characteristics [6]. The latent representations of users along with items can be used to estimate future ratings or calculate missing ratings once the matrix has been factorized.

In network analysis, community detection stands as a fundamental task, endeavoring to identify cohesive subsets of nodes, referred to as communities or modules within a network [7]. These communities in networks signify groups of nodes that display stronger interconnections amongst themselves compared to connections with nodes outside the community [8]. These communities provide insightful information on the structure, organization, and dynamics of complex systems [9,10]. The applications of community detection across various domains, such as social network analysis, biological networks, online forums, and recommendation systems, have garnered substantial attention [11]. Through the identification of communities, we gain insights into the underlying interaction patterns, discover influential groups, and develop a deeper comprehension of the network’s functionality [12].

### 1.1. Applications

Matrix factorization techniques have been widely applied across diverse domains, demonstrating their versatility and effectiveness. Some of the notable applications of the matrix factorization techniques include

1. **Natural Language Processing (NLP)**: Within the field of NLP, techniques for matrix factorization have been used in a variety of tasks in topic modeling, text classification, etc. [13,14]. Through the decomposition of the document matrix, MF algorithms can reveal latent representations that effectively capture the underlying semantic structure of the textual data.
2. **Social Network Analysis**: MF techniques have been utilized in social network analysis to unveil communities of individuals sharing common interests or behaviors [15,16]. SVD++ facilitates the identification of friends, influencers, or interest-based communities by enhancing user experience and engagement on social media platforms. FANMF is used to uncover community structures and identify influential nodes within the network. FANMF can effectively detect the group of nodes by exposing hidden relationships and structures by factorizing them into nonnegative adjacency matrices.
3. **E-commerce**: In the realm of e-commerce platforms, SVD++ is extensively utilized to deliver personalized product recommendations to users [17,18]. By including implicit feedback, supplementary data, and user-item ratings, SVD++ can adeptly capture user preferences and item characteristics for accurate product recommendations [19].
4. **Streaming Services**: Streaming platforms, including music or video services or SVD++, provide personalized content recommendations to the users. SVD++ significantly enhances the discovery and recommendation of relevant and captivating content. It ensures that the users are presented with content aligned with their individual tastes and preferences [20].
5. **Image Processing**: FANMF finds application in image processing tasks, where the image data are factored into nonnegative matrices. By extraction, it improves image quality and facilitates the analysis of visual data. By decomposition, matrix factorization algorithms are capable of distinguishing noise from the underlying structure. It also completes missing parts and extracts significant features for analysis and representation [21].
6. **Nutritional Recommendation**: In the realm of nutritional recommendations, matrix factorization entails structuring dietary information into a user-item matrix. This matrix uncovers hidden factors linked to individual tastes and nutritional traits, facilitating the delivery of personalized dietary advice. By accounting for variables such as taste preferences, dietary constraints, and health objectives, this approach aids individuals in devising well-balanced diets, promoting healthier and custom-tailored eating habits [22].

In essence, the applications of MF techniques have a broad scope of transforming the methods through which we analyze, comprehend, and leverage complex data [23]. The ongoing evolution of these techniques holds the potential to unlock fresh possibilities and propel advancements in numerous domains. It ultimately benefits individuals, organizations, and society at large.

### 1.2. Problem Statement

Schafer et al. [24] describe the problem of recommender systems as follows:

In the context of a set of users, denoted as *U* = $\left\{u_{1},u_{2},\ldots ,u_{a}\right\}$, a set of items or products, denoted as *I* = $\left\{I_{1},I_{2},\ldots ,I_{b}\right\}$, and the corresponding user-item ratings, where $R_{ij}$ represents the rating assigned by user $u_{i}$ to item $I_{j}$, the primary objective of recommender systems is to suggest a new item $I_{j}$ to a user $u_{i}$ that the user has not yet encountered or consumed.

**Figure 1:**
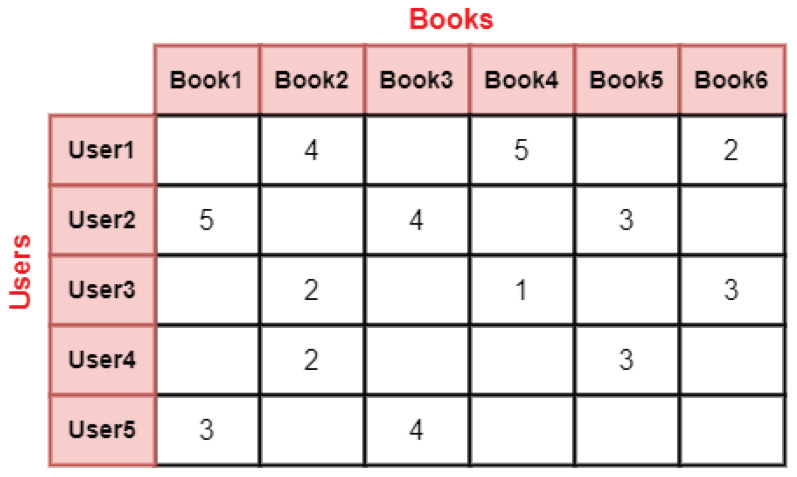
An example of simple rating matrix.

As an example, let us take a look at the matrix depicted in Figure 1. In this matrix, each row corresponds to a user, and each column represents an item, such as books. The specific value $R_{ij}$ within the matrix indicates the ratings provided by user $u_{i}$ for item $I_{j}$. The main objective of recommender systems lies in forecasting the values within the rating matrix that have not yet been assigned or rated.

Content-based recommendation and collaborative filtering represent two widely adopted approaches for solving the problem of recommender systems. Matrix factorization (MF), is a prevalent model-based collaborative filtering technique. The main challenge with MF is its computational intensity. The objective of this study is to enhance the recommendation quality while concurrently reducing the time complexity associated with MF techniques. To achieve this goal, we propose representing the rating matrix as a complex network. Complex networks inherently manifest a community structure. We aim to leverage the community information in order to parallelize the matrix factorization technique.

**Figure 2:**
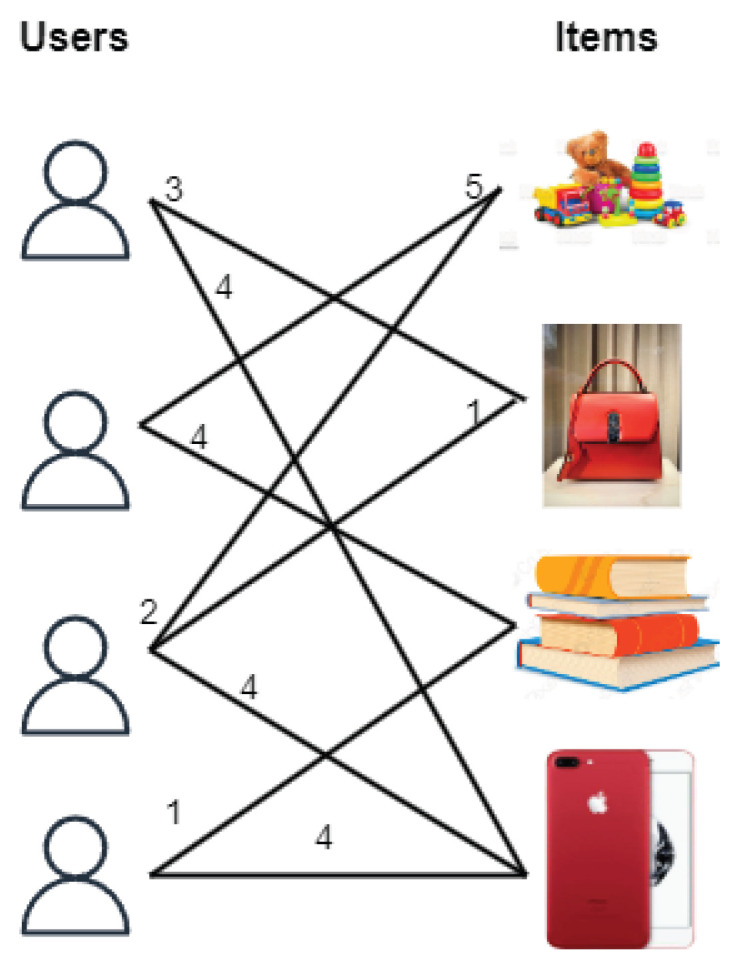
An example of a user-item bipartite network with edges denoting a rating given by the user for the item.

Recommender systems are natural instances of weighted complex bipartite networks. A bipartite network is characterized by having exactly two distinct node types and the presence of edges connecting nodes of different types. More formally, a bipartite network is defined as *G* = $\left(V_{1}\cup V_{2},\;E\right)$, where $V_{1}$ and $V_{2}$ denote two sets of node types, and *E* represents the set of edges connecting nodes from $V_{1}$ to nodes from $V_{2}$. You can observe an illustrative example of this in Figure 2, which represents a scenario where users make purchases on e-commerce platforms like Amazon, portraying the interaction as a bipartite network.

We propose that the community information available in the network can be successfully incorporated into MF techniques. This approach not only enhances the quality of recommendations but also provides a parallel framework based on community division to the MF technique, which addresses the problem of computational complexity of MF. This approach requires MF techniques as well as community detection algorithms. The following section briefly reviews the two.

## 2. Literature Review

In recent years, the MF method has garnered significant attention as a widely adopted and successful method for rating prediction in recommendation systems. The Netflix Prize competition, which was started in 2006, is one early piece of work noteworthy in relation to MF in recommender systems [25]. The fundamental matrix factorization model creates user and item latent feature matrices from the rating matrix, enhancing the accuracy of rating predictions through the understanding of possible connections between users and items.

As the information interconnection era has emerged, the basic MF model no longer satisfies the demands of recommender systems. It thus leads to the emergence of numerous variants of this model. Excluding all the nonnegative entries in latent features, a model nonnegative matrix factorization (NMF) was initiated by Paatero and Tapper in 1994 that improves the accuracy of the model [26]. Mnih et al. in the year 2007 proposed probabilistic matrix factorization (PMF), which utilizes probabilistic modeling to effectively capture uncertainties in user-item ratings, resulting in recommendations that are more reliable and precise [27]. The significance of the singular value decomposition (SVD) technique was introduced by Mastorakis in 1857 and researchers started to apply SVD to the recommendation domain from 2006 [28]. The method has the ability to perform robust MF, enabling the identification of hidden features and effective dimensionality reduction in data analysis.

By integrating explicit and implicit feedback to enhance recommendation accuracy and personalization, Koren et al. in the year 2008 introduced an advanced singular value decomposition (SVD++) method [29]. Through the incorporation of user-item ratings and implicit feedback, SVD++ boosts the performance of the recommender systems, enabling better capture of user preferences and more effective recommendation generation. In 2015, Shi et al. introduced a method named pairwisely constrained nonnegative symmetric matrix factorization (PCSNMF) that incorporates the symmetric community structures found in undirected networks but also leverages pairwise constraints derived from ground-truth group information [30]. In 2017, deep matrix factorization (DMF) was introduced by Xiangnan et al. and the deep learning with MF [31] emerges. This method enables the discovery of intricate patterns and the extraction of complex features from large-scale datasets. Kipf and Welling in the year 2018 combined the power of MF and graph convolutional neural networks to capture both collaborative filtering patterns and graph structures in recommendation systems [32]. By incorporating the graph information, the recommendation accuracy is improved by leveraging the connectivity and relationships among users and items. In 2019, factorized asymmetric nonnegative matrix factorization (FANMF) was introduced by Tosyali et al. by considering the asymmetric relationships between users and items [33]. It led to enhanced recommendation quality, a deeper understanding of user preferences, and ultimately provided personalized user experiences in various data analysis tasks.

Community detection is another concept used by our proposed approach. The existing community detection algorithms are reviewed here. The Girvan–Neuman algorithm uses edge betweenness centrality to iteratively remove edges and identify communities in a network [34]. In 2007, Raghavan et al. developed a label propagation algorithm. The label propagation algorithm updates the node labels iteratively based on the majority of their neighbors’ community labels to effectively detect communities in a network  [35]. Modularity is one of the metrics used for evaluating the quality of community detection. It assesses the extent to which a network can be partitioned into discrete communities or modules by analyzing the connectivity patterns between nodes. The Louvain algorithm was proposed by Blondel et al. The Louvain algorithm has the ability to efficiently optimize modularity, making it highly effective in detecting communities in large-scale networks while maintaining computational speed [36]. In 2011, Pons and Latapy introduced the Walktrap algorithm, which is notable for its use of random walks to assess node similarities for locating communities in large networks [37]. The Leiden algorithm was developed in 2019 by Traag et al. with iterative application results in a partition when local best assignments are made to all community subsets  [38]. The following section briefly discusses the MF techniques and community detection algorithm chosen in our experimentation highlighting the cause of selection.

## 3. Methodology

In this work, we propose a parallel framework for matrix factorization. This section first provides an elaborate exposition elucidating the various matrix factorization methods, and then discusses the community detection algorithms.

### 3.1. Matrix Factorization

#### 3.1.1. Basic Matrix Factorization

Simon Funk’s work during the Netflix Prize competition in 2006 played a significant role in popularizing the use of matrix factorization algorithms for recommender systems [25].

Given a rating matrix *R* of size a×b representing ratings given by *a* users on *b* items, where a huge number of ratings are unknown, the matrix factorization technique performs the following five steps.

- Step 1: Initialize the entries of user latent feature matrix *M* and item latent feature matrix *N* of sizes a×k and b×k, respectively, with random values. *k* is the number of latent features, tuned experimentally with different values of *k*.
- Step 2: Multiply the matrices *M* and *N* to obtain the predicted rating matrix with non-empty cells having some predicted ratings, as shown below in Equation (1).
  (1) $$\tilde{R}=MN^{T}$$
  $$\left[\begin{array}{ccc} \tilde{r_{11}} & .. & \tilde{r_{1b}}\\ . & .. & .\\ . & .. & .\\ . & .. & .\\ \tilde{r_{a1}} & .. & \tilde{r_{ab}} \end{array}\right]=\left[\begin{array}{ccc} m_{11} & .. & m_{1k}\\ . & .. & .\\ . & .. & .\\ . & .. & .\\ m_{a1} & .. & m_{ak} \end{array}\right]\left[\begin{array}{ccc} n_{11} & .. & n_{1b}\\ . & .. & .\\ . & .. & .\\ . & .. & .\\ n_{k1} & .. & n_{kb} \end{array}\right]$$
                                      a×b                a×k            k×b
  Note that the given rating matrix *R* is approximately equal to $MN^{T}$ as shown in Equation (2).
  (2) $$R\approx MN^{T}$$
- Step 3: Compute the deviation between actual and predicted ratings as shown in Equation (3), where $m_{a}$ is of order $m_{a\times k}$ and $n_{b}$ is of order $n_{b\times k}$.
  (3) $$r_{ab}\approx m_{a}n_{b}^{T}$$
- Step 4: Minimize the error in the prediction. It is common to use Equation (4) to compute the squared error.
  (4) $$min\sum_{a,b}{\left(r_{ab}-m_{a}n_{b}^{T}\right)}^{2}$$
  In order to avoid overfitting the squared error, the regularization term is added as shown in Equation (5).
  (5) $$min\sum_{a,b}{\left(r_{ab}-m_{a}n_{b}^{T}\right)}^{2}+\alpha (||m_{a}{\left|\right|}^{2}+\left|\right|n_{b}{\left|\right|}^{2})$$
  The impact of the regularization is controlled by a constant α. ||.|| is the frobenius norm. The approximation of this value is calculated using stochastic gradient descent or alternating least squares. For each rating in the training data, the prediction error is calculated using the stochastic gradient descent method as displayed in Equation (6).
  (6) $$e_{ab}=r_{ab}-m_{a}n_{b}^{T}$$
- Step 5: The following update rules shown in Equation (7) are used to update the matrices *M* and *N* to minimize squared error.
  (7) $$\begin{array}{c} n_{b}⟵n_{b}+\beta \left(e_{ab}m_{a}-\alpha n_{b}\right)\\ m_{a}⟵m_{a}+\beta \left(e_{ab}n_{b}-\alpha m_{a}\right) \end{array}$$
  The representation of a matrix for the above Equation (7) are as follows:
  For the equation
  $$\begin{array}{c} n_{b}⟵n_{b}+\beta \left(e_{ab}m_{a}-\alpha n_{b}\right) \end{array}$$
  the matrix representation is
  $$\left[\begin{array}{ccc} n_{11} & .. & n_{1b}\\ . & .. & .\\ . & .. & .\\ . & .. & .\\ n_{k1} & .. & n_{kb} \end{array}\right]⟵\left[\begin{array}{ccc} n_{11} & .. & n_{1b}\\ . & .. & .\\ . & .. & .\\ . & .. & .\\ n_{k1} & .. & n_{kb} \end{array}\right]+\beta e_{ab}\left[\begin{array}{ccc} m_{11} & .. & m_{1k}\\ . & .. & .\\ . & .. & .\\ . & .. & .\\ m_{a1} & .. & m_{ak} \end{array}\right]-\beta \alpha \left[\begin{array}{ccc} n_{11} & .. & n_{1b}\\ . & .. & .\\ . & .. & .\\ . & .. & .\\ n_{k1} & .. & n_{kb} \end{array}\right]$$
  For the equation
  $$\begin{array}{c} m_{a}⟵m_{a}+\beta \left(e_{ab}n_{b}-\alpha m_{a}\right) \end{array}$$
  the matrix representation is
  $$\left[\begin{array}{ccc} m_{11} & .. & m_{1k}\\ . & .. & .\\ . & .. & .\\ . & .. & .\\ m_{k1} & .. & m_{ak} \end{array}\right]⟵\left[\begin{array}{ccc} m_{11} & .. & m_{1k}\\ . & .. & .\\ . & .. & .\\ . & .. & .\\ m_{k1} & .. & m_{ak} \end{array}\right]+\beta e_{ab}\left[\begin{array}{ccc} n_{11} & .. & n_{1b}\\ . & .. & .\\ . & .. & .\\ . & .. & .\\ n_{k1} & .. & n_{kb} \end{array}\right]-\beta \alpha \left[\begin{array}{ccc} m_{11} & .. & m_{1k}\\ . & .. & .\\ . & .. & .\\ . & .. & .\\ m_{a1} & .. & m_{ak} \end{array}\right]$$
- The steps 3, 4, and 5 are repeated until either the number of iterations is fixed or the error reaches 0.

This approach has a time complexity of O(abk), where a,b are the number of users and items, and *k* is the number of latent features.

#### 3.1.2. SVD++

SVD++ is an extension of the singular value decomposition method  [29].

In the SVD++ method, implicit feedback is added to the user’s latent feature vector [39,40]. The implicit feedback is computed in the form of two matrices, user feedback matrix *P* and item feedback matrix *Q*. The user feedback matrix *P* is a matrix of the same size as *R*. At first, the value of $P=[p_{ab}]$$\forall \left(u_{a},I_{b}\right)$ is set to be 1 if $r_{ab}$ is observed; it is 0 otherwise. Later, the entries of *P* are normalized row-wise as follows. Let $I_{j}$ be the item that the user $u_{i}$ has rated; each nonzero entry in the $j^{th}$ row of *P* is computed as $\frac{1}{\sqrt{|I_{j}|}}$. Matrix *Q* is the same as the item latent feature vector of order b×k. Once the feedback matrices *P* and *Q* are available, the dot product of *P* and *Q* matrices is computed and added to the user latent feature matrix. This user latent feature matrix computed is used in the procedure of SVD++ by adding an implicit feedback matrix. Then, the deviation between the original rating matrix R, i.e., ($m_{a}n_{b})$, and the predicted rating matrix $\tilde{R}$, i.e., (${\left(m+PQ\right)}_{a}n_{b}$), is computed as the RMSE value as shown in Equation (8).

The time complexity for the SVD++ method is O(abk), where a,b are the count of users and items, *k* is the number of latent features.

#### 3.1.3. Factorized Asymmetric nonnegative Matrix Factorization (FANMF)

In 2019, the FANMF method was introduced by Tosyali [33]. The main purpose of the FANMF method is to handle nonnegative data that have an asymmetric nature. Data in the real world are often asymmetrical, where the rows and columns are related in this way and are not symmetrical [41]. In the FANMF method, a rating matrix is decomposed into two nonnegative latent feature matrices. FANMF is different from the traditional methods, as it accommodates the feature matrices of different dimensions that enable us to capture the data that are asymmetric in nature. It also adds a sparsity constraint along with the nonnegativity constraint, which contains only a limited number of nonzero elements.

FANMF extends NMF to asymmetric scenarios where the user and item bias are considered in addition to the user-item interactions to make the model more accurate and improve the recommendation quality. User item bias includes the inherent inclinations of users towards specific items or the inherent attractiveness of items to users, independent of their previous interactions or behaviors.

The original rating is represented as the product of user and item rating vectors as shown in Equation (3). Update the values of *M* and *N* by using a multiplicative update algorithm that contains update values [42].

$$\begin{array}{ccc} M & = & M\cdot \times (\left(R\cdot /\left(M\times N+\left(R==0\right)\right)\right)\times N^{T})\\ N & = & N\cdot \times (M^{T}\times \left(R\cdot /\left(M\times N+\left(R==0\right)\right)\right)) \end{array}$$

where M·×N is the dot product of *M* and *N* similar to M·/*N* is the dot division of *M* and *N*, i.e., element wise division. M×N is the product of two matrices *M* and *N*. $M^{T}$ is the transpose of the matrix *M*. The term R==0 is included in the denominator to prevent division by zero.

Make the dot product of the updated latent feature matrices, which is $\tilde{R}$. The time complexity for the matrix factorization method is given as O(abk), where a,b are the number of users and items, and *k* is the number of features that were extracted.

After computing the predicted rating matrix using any of the methods specified above, the error in prediction is computed using root mean square error (RMSE) given in Equation (8).

(8) $$RMSE=\sqrt{\frac{1}{N}\sum {\left(r_{ab}-{\tilde{r}}_{ab}\right)}^{2}}$$

where $r_{ab}$ is the original rating, ${\tilde{r}}_{ab}$ is the predicted rating, and *N* is the total number of predictions.

In this work, we introduce parallelism in matrix factorization with the help of community information. Various community detection algorithms are available in the literature to divide a complex network into communities.

### 3.2. Community Detection

The Girvan–Newman algorithm is one of the first popular algorithms that uses edge betweenness centrality to detect communities in networks, achieved through iterative removal of edges with high betweenness. The shortcoming of the above-mentioned algorithm is while it excels at identifying overlapping communities, it faces the drawback of being computationally expensive, particularly for large graphs, due to repeated edge removals and recalculations of betweenness. The Louvain algorithm addresses these shortcomings by adopting an efficient approach centered around optimizing modularity. Through iterative modularity optimization with node movements, it achieves fast and scalable community detection, effectively capturing communities even in large-scale networks. Louvain’s superior optimization of modularity enables it to outperform Girvan–Newman in terms of speed and scalability, making it the preferred choice for real-world community detection tasks involving massive and complex networks. Therefore, we use the Louvain algorithm to divide the rating network into communities. The next subsection gives the details of Louvain.

#### Louvain Algorithm

The Louvain algorithm works effectively in covering large communities or groups that are densely interconnected in a network. It employs a modularity optimization process, iteratively refining the network’s community structure to maximize modularity.

The Louvain algorithm has a six-step procedure to find the appropriate communities. Each node in the initial step is attached to its own community. Next, rearrange the nodes between the communities that iteratively optimize the modularity score, which elevates the standards of the community structure. By iteratively considering each node, evaluate the modularity score by relocating the nodes to nearby communities. It is followed until there is no change in the modularity score. A new graph is constructed by combining the nodes in each community by connecting the nodes and edges of different communities. All these steps are repeated until there is no change in the modularity score observed.

The Louvain algorithm has become popular because of its efficiency in detecting communities in the network by achieving a high modularity score, all while maintaining computational efficiency [9]. A higher modularity score signifies a more robust community structure, characterized by tightly interconnected nodes within communities while having fewer connections between communities [43,44]. The calculation of modularity score is shown in Equation (9). This indicates the algorithm’s successful identification of distinct and internally cohesive communities, demonstrating their meaningful organization within the network. The Louvain community detection method takes O(nlogn) time complexity, where *n* is the graph’s number of nodes.

In community detection algorithms, modularity plays a significant impact in determining the standard of the communities that are formed. Girvan and Newman introduced the modularity measure to assess the standards of the communities that are formed and defined modularity as shown in Equation (9) [34].

(9) $$G=\frac{1}{2m}\sum_{\left(i,j\right)}\left[A_{ij}-\frac{k_{i}k_{j}}{2m}\right]\delta \left(c_{i},c_{j}\right)$$

where *m* is the total number of edges in the graph, $A_{ij}$ is the edge weight between the nodes *i* and *j*, $k_{i}$ and $k_{j}$ are the degree of nodes *i* and *j*, respectively, $c_{i},c_{j}$ are the communities to which nodes *i* and *j* belong, and $\delta (c_{i},c_{j})$ is the Kronecker delta function which is equal to 1 if $c_{i}=c_{j}$ and 0 otherwise. The term $\left[A_{ij}-\frac{k_{i}k_{j}}{2m}\right]$ represents the difference between the observed number of edges between the nodes *i* and *j* and the expected number of edges under a null model where edges are placed at random. The values of modularity (G) vary between −1 and +1.

## 4. Proposed Method

In this section, a brief description of the motivation behind the proposed method and in detail about the Community-Based Matrix Factorization (CBMF) approach.

### 4.1. Motivation

In any type of MF method, the rating matrix is constructed by using users and items. The predicted rating matrix is obtained by using different update rules for the latent feature matrices in each kind of MF. The variation between the original and the predicted rating matrix is evaluated using root mean square error. The matrix factorization method predicts a rating for every user item combination. However, there are some cases such that a user may never consume a set of products. Because MF predicts a rating for them, they are recommended to users. This creates a lot of false recommendations. There are only a limited group of items that a user may consume. We propose that by modeling the matrix into a bipartite network containing user and item nodes, users and items can be clustered into tight communities using community detection algorithms. By limiting MF to only the communities, the prediction of false recommendations will significantly reduce. This approach also reduces the time complexity of MF methods.

### 4.2. Community-Based Matrix Factorization (CBMF) Approach

With this motivation, we propose a Community-Based Matrix Factorization (CBMF) approach, which is given in Algorithm 1.
**Algorithm 1:** Community-Based Matrix Factorization (CBMF)
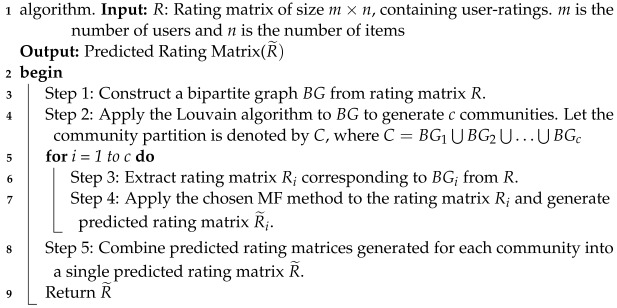


**Figure 3:**
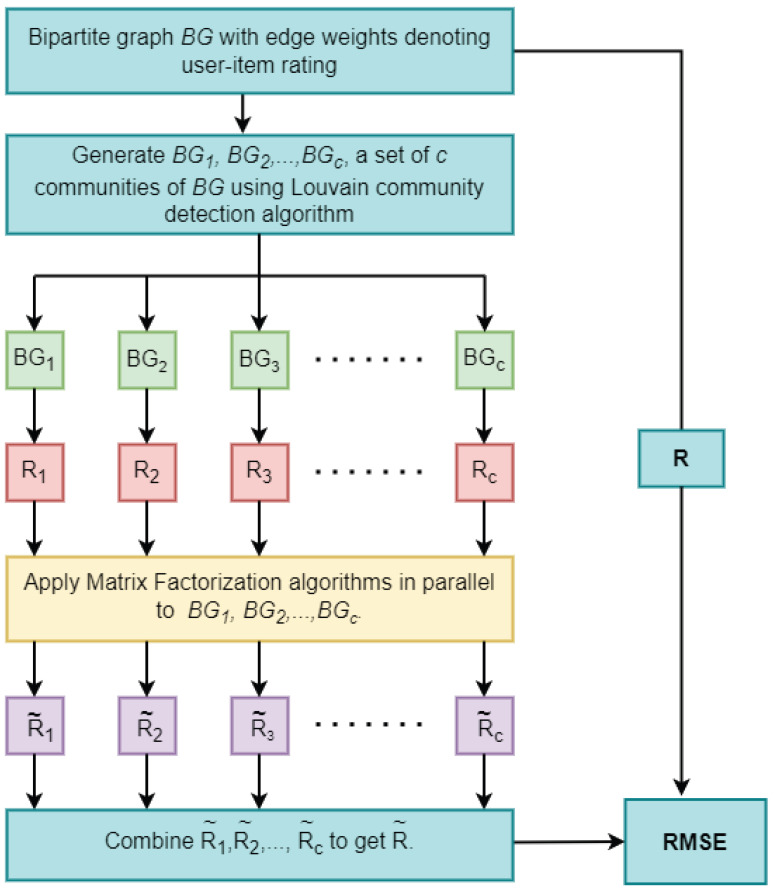
Community-Based Matrix Factorization (CBMF) framework.

The proposed CBMF constructs a bipartite graph BG containing user and item nodes. The bipartite graph contains a node for each user as well as an item. An edge is formed from the user node to the item node if the user rates the item. The Louvain community detection algorithm is applied on the constructed bipartite graph to divide into communities. Extract rating matrices pertaining only to the nodes in the communities. Note that these rating matrices are very small in size compared to the original rating matrix. Now, apply the MF method to each of these smaller rating matrices in parallel. Combine all the predicted rating matrices into a single prediction rating matrix. Compute RMSE between the original rating matrix and the predicted by the CBMF approach. The procedure is depicted in Figure 3.

### 4.3. Time Complexity

For the MF method, let a×b be a rating matrix of *a* users, *b* items, and *k* latent features. The iterations are the same for every method and are taken as constant. The time complexity required for calculating the MF method is typically considered to be O(abk) [45]. The Louvain community detection method takes O(nlogn) time complexity, where *n* is the number of nodes of graph [46].

The bipartite graph BG containing users and items is given as input for the Louvain algorithm, with the nodes as users and items. Here, in this case, (a+b) will be the nodes in the graph. Therefore, the time complexity of the Louvain algorithm will be O((a+b)log(a+b)). A bipartite graph BG is constructed from and is divided into appropriate community structures by using the Louvain algorithm (say $c_{1},c_{2},\cdots ,c_{c}$). From each community, a small rating matrix will be obtained (say $R_{1},R_{2},\cdots ,R_{c}$). Apply MF time complexity for each rating matrix and we get a time complexity of $O(a_{1}b_{1}k_{1})$, $O(a_{2}b_{2}k_{2})$,⋯, $O(a_{c}b_{c}k_{c})$; consider the maximum of all these time complexities. Suppose $R_{p}$ community rating matrix has the maximum value and the time complexity is $O(a_{p}b_{p}k_{p})$.

Hence, the overall time complexity for the MF method using the Louvain community detection method is O((a+b)log(a+b))+$O(a_{p}b_{p}k_{p})$.

Similarly for the SVD++ and FANMF methods, the time complexity is O(abk). Therefore, the total time complexity that is required for the calculation of MF, SVD++, and FANMF methods will be O((a+b)log(a+b))+$O(a_{p}b_{p}k_{p})$.

## 5. Dataset Statistics

We have evaluated the approach of CBMF on different datasets from different domains. Datasets, namely MovieLens 100K, Film Trust, Jester, Good Books, Wikilens, and Cell Phone Recommendation, are taken. The dataset statistics of all these datasets are shown in Table 1. From all the six datasets we have taken, datasets, namely MovieLens 100K [47], Good Books [48], and Cell Phone Recommendation [49], are taken from the Kaggle repository. The other three datasets, namely Film Trust [50], Jester [51], and Wikilens [52], are taken from the Konect repository.

**Figure 4:**
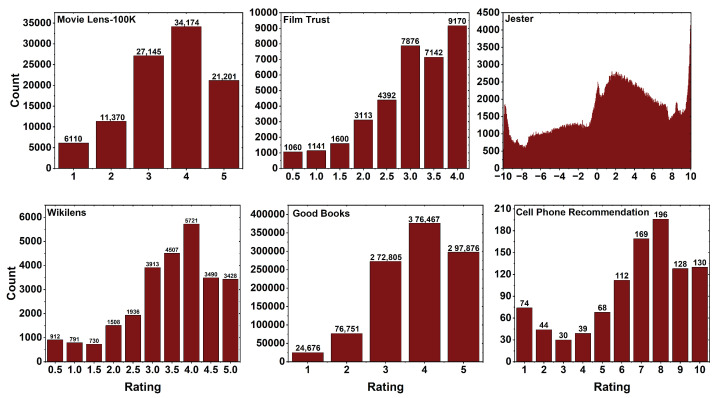
Rating distribution plots for six different datasets, namely MovieLens 100K, Film Trust, Jester, Wikilens, Good Books, and Cell Phone Recommendation.

All of these datasets’ distribution plots are displayed in Figure 4. The plots are drawn by taking the ratings; the X-axis represents users, while the Y-axis represents the count of each rating provided by those users. In the dataset MovieLens 100K, the users are the people and the items are the movies in the dataset. The rating distribution is from 1 to 5 where the people have rated movies. It is observed in the plot that 34,174 of the users had given a higher rating of four for the movies, and a low rating of one is given by 6110 the users out of 100,000 ratings. For the dataset Film Trust, the users are the people and the items are the films having a rating distribution from 0.5 to 4 where the people have rated films. It is observed in the plot that 9170 users had given a rating of five for films, and a low rating of 0.5 is given by the users 1060 out of 35,494 ratings.

In the dataset Jester, the users are the people, and the items are jokes having a rating distribution from −10 to +10 where the people have rated the jokes. It is observed that there are above 4000 users who have rated +10 in the dataset out of 1,048,575 ratings.

The Wikilens dataset is shown in Figure 4, where the users are the people and the items are the Wikipedia articles. The rating distribution is given from 0.5 to 5, where the users rated the items in Wikipedia. From the plot, it is observed that a higher rating of four is given by 5721 users, and a low rating of 1.5 is given by 730 users out of 26,936 ratings. In the dataset Good Books, people are the users, and the items are the books having a rating distribution from 1 to 5. The rating is given by the users on different books. From the plot, we can observe that 376,467 of the users have given a higher rating of four for the books, and a low rating of one is given by 24,676 users out of 1,048,575 ratings. For the Cell Phone Recommendation dataset, the users are the people and the items are the cell phone ids having a rating distribution from 1 to 10. The ratings are given by people for different cell phone ids. From the plot, it is observed that a higher rating of eight is given by 196 users, and a low rating of three is given by 30 users out of 990 ratings.

**Figure 5:**
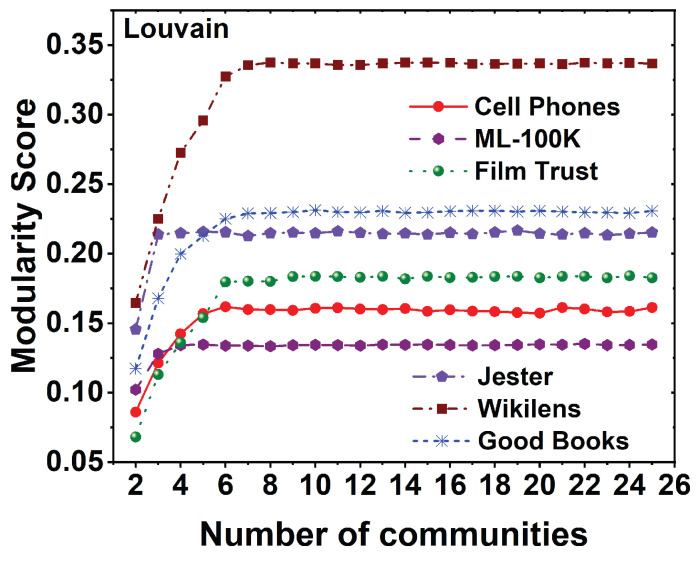
Modularity performance for six different datasets, namely MovieLens 100K, Film Trust, Jester, Wikilens, Good Books, and Cell Phone Recommendation for the Louvain algorithm.

As community detection is a major part of CBMF, we evaluate the modularity of different datasets using the Louvain algorithm for different communities. The modularity of six different datasets using 25 communities is shown in Figure 5. It is observed from all the datasets that the modularity score is increasing as the communities are increased and maintained constant after a certain number of communities. The highest modularity score is maintained by the Wikilens dataset, and the lowest modularity score is given by the MovieLens 100K dataset. The time taken for calculating the modularity score is shown in Table 2. It is observed that the Good Books dataset takes more time to divide the data into 25 communities, and the least time is taken by the Cell Phone Recommendation dataset. The time taken to execute depends on the size of the dataset and the implementation of the computational experiment.

The specifics regarding the hardware and software specifications employed in our implementation are as follows: The system boasts impressive specifications, featuring an 11th Gen Intel Core i9 processor clocked at 2.5 GHz, a formidable 64 GB of RAM, and a 64-bit operating system. Furthermore, Python is the programming language, renowned for its simplicity and effectiveness in a wide array of programming and data analysis tasks.

## 6. Result Analysis

This section presents a series of experiments designed to demonstrate the validity of the hypothesis proposed in the paper. Using the Louvain community detection algorithm, three different matrix factorization methods (MF, SVD++, and FANMF) are employed to predict the missing entries in the rating matrix across six distinct datasets as shown in Table 1.

The RMSE results of CBMF against their non-community variations for three MF techniques on six datasets are tabulated in Table 3.

Because the best number of communities is not uniform for all networks, Table 3 contains the RMSE results of CBMF where the networks show high modularity in community division using the Louvain algorithm. For instance, MovieLens 100K, Film Trust, and Jester networks have the best modularity when they are divided into 25 communities. Good Books shows high modularity when divided into 20 communities, and Wikilens and Cell Phone when divided into 15 communities. In each network, for each MF technique, CBMF outperformed the non-CBMF technique. Further experimental results are given in detail below.

The computation of the root mean square error (RMSE) as shown in Equation (8) involves varying the number of latent features at values of *k* as 2,10,20,30,50. Considering the random nature of the Louvain algorithm, each MF method is iterated for 25 communities and 25 iterations. The evaluation of different patterns in RMSE values, along with the total time taken to assess the MF method for six datasets, is presented. The resulting graph depicts the relationship between the number of communities on the X-axis and the corresponding RMSE value on the Y-axis. Given that the Cell Phone Recommendation dataset comprises only 99 users and 33 items, the value of *k* should be the minimum of number of users and items. Therefore, the iteration for *k* is limited to 30.

**Figure 6:**
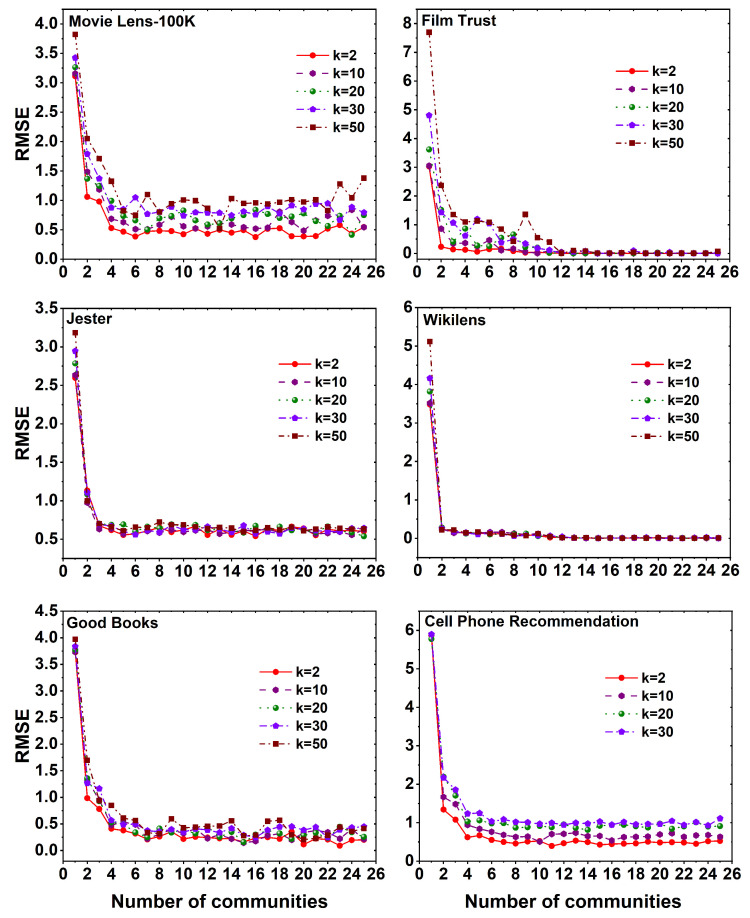
RMSE plots using MF method for six different datasets, namely MovieLens 100K, Film Trust, Jester, Wikilens, Good Books, and Cell Phone Recommendation with 25 communities and different *k* (latent features) values.

Figure 6 illustrates the variation in RMSE values across six distinct datasets when employing the MF method with different *k* values on a set of 25 communities. A notable observation from the graph is that, in all datasets, the absence of the Louvain algorithm results in the highest RMSE value when *c* is 1. However, as the number of communities increases through the utilization of the Louvain algorithm, i.e., *c* values range from 2,3,⋯,25, a decline in the RMSE value is observed. Notably, the MovieLens 100K, Good Books, and Cell Phone Recommendation datasets exhibit an increase in the RMSE value with higher *k* values. Conversely, in the Film Trust and Jester datasets, the RMSE value stabilizes after reaching a certain number of communities, regardless of the *k* value. Furthermore, in the Wikilens dataset, there exhibit a constant relationship between the RMSE value for different communities as well as the *k* values. Overall, it is evident that the MF method consistently yields better RMSE values with lower *k* values across all datasets.

**Figure 7:**
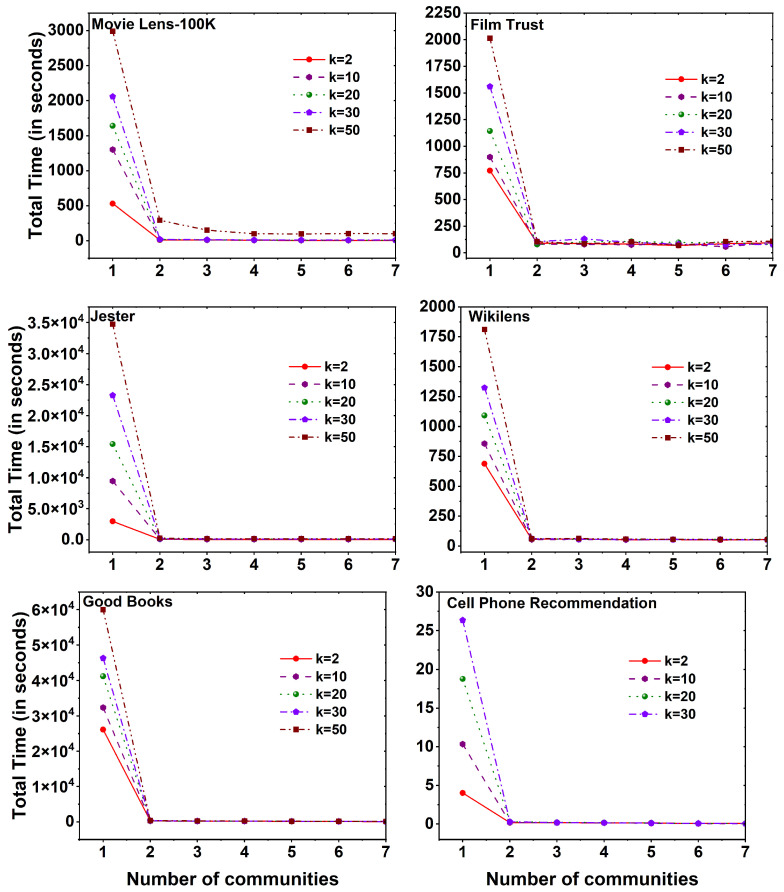
Time taken to calculate MF method for six different datasets, namely MovieLens 100K, Film Trust, Jester, Wikilens, Good Books, and Cell Phone Recommendation with 25 communities.

Figure 7 displays the computational time required to execute the MF method on six distinct datasets, employing different *k* values across a set of 25 communities. Across all datasets, a noticeable observation is that the computation time for the MF method, without the utilization of community division, exceeds that of the method with community division. As the number of communities increases, a reduction in computational time is observed. Interestingly, the evaluation time of the method remains constant as the number of communities expands, regardless of the specific *k* values employed.

**Figure 8:**
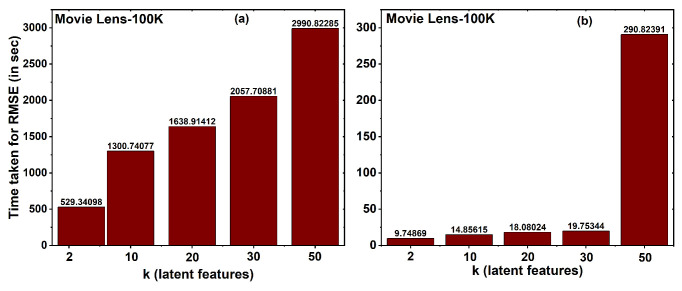
(**a**) Time taken to calculate RMSE for community 1 using non-CBMF approach for MF method in MovieLens 100K dataset. (**b**) Time taken to calculate RMSE for community 2 using CBMF approach for MF method in MovieLens 100K dataset.

Figure 8 depicts the computational time required to calculate the root mean square error (RMSE) for the MovieLens 100K dataset when employing MF with both Non-Community-Based Matrix Factorization (Non-CBMF) and Community-Based Matrix Factorization (CBMF) methodologies. It is evident from the figure that RMSE computation without employing the CBMF approach consumes a significant amount of time. For the case of *k* value 2 latent features, the RMSE calculation takes approximately 529.34 s. In contrast, when utilizing the CBMF approach with the same number of latent features (for *k* value 2), the time required is significantly reduced to a mere 9.74 seconds. This marked contrast in computation time becomes even more pronounced as the number of latent features increases. Moreover, an analogous trend is observed as the number of communities increases, resulting in an extended RMSE computation time.

**Figure 9:**
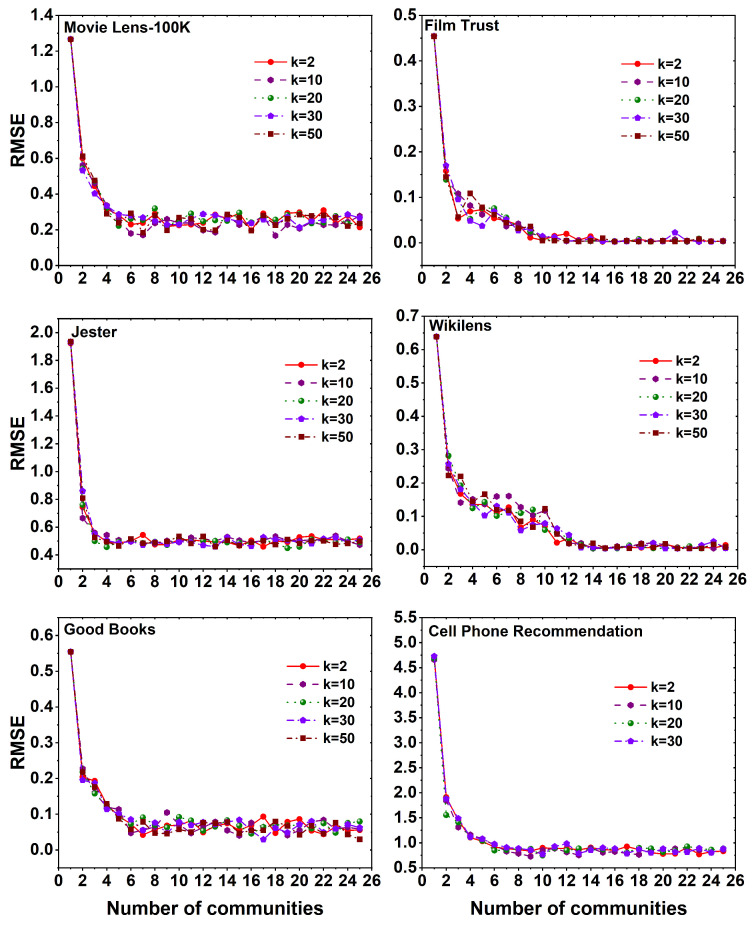
RMSE plots using SVD++ method for six different datasets, namely MovieLens 100K, Film Trust, Jester, Wikilens, Good Books, and Cell Phone Recommendation with 25 communities.

Figure 9 presents the RMSE values across six distinct datasets when employing the SVD++ method with varying *k* values on a set of 25 communities. Notably, at community 1, a significantly high RMSE value is observed across all datasets. However, as the communities are divided, a decline in the RMSE value is observed, reaching a stable state after a certain number of communities. It is worth noting that in the SVD++ method, subtle variations in the RMSE value can be observed for different *k* values across all datasets.

**Figure 10:**
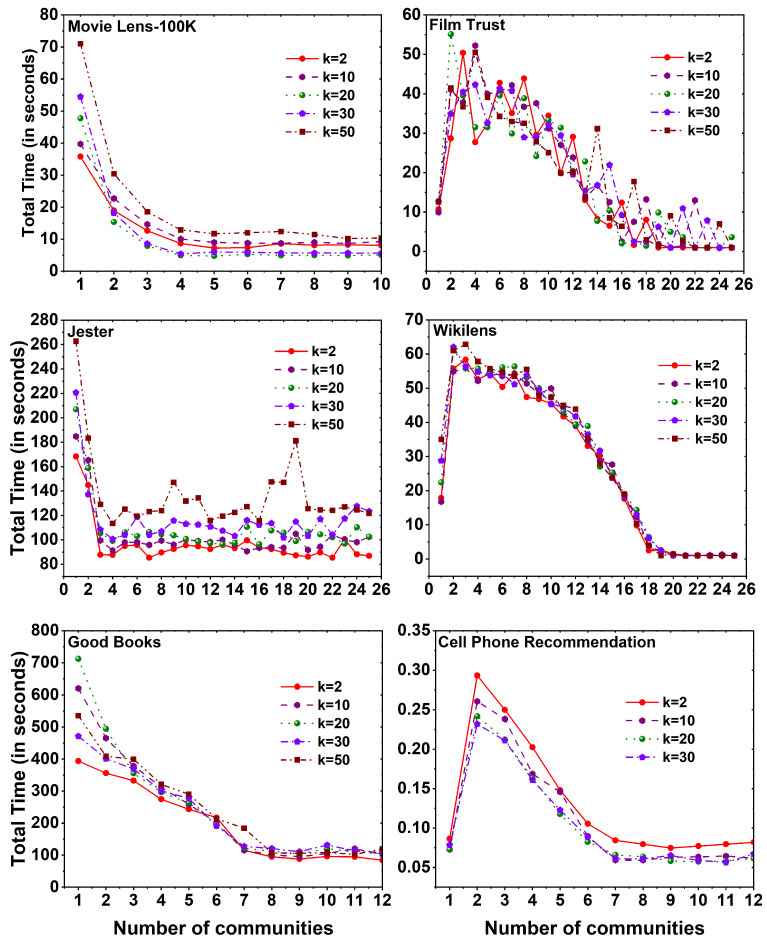
Time taken to calculate SVD++ method for six different datasets, namely MovieLens 100K, Film Trust, Jester, Wikilens, Good Books, and Cell Phone Recommendation with 25 communities.

Figure 10 displays the computational time required to execute the SVD++ method on six distinct datasets, employing different *k* values across a set of 25 communities. Noteworthy observations can be made across various datasets. In the MovieLens 100K and Good Books datasets, an increase in the number of communities leads to a decrease in the evaluation time of the method. The maximum evaluation time is observed at community 1. Conversely, in the Jester dataset, the maximum time is taken to evaluate the SVD++ method at *c* value 1, with slight fluctuations in time as the communities increase. Furthermore, for the Film Trust, Wikilens, and Cell Phone Recommendation datasets, it is observed that initially, evaluating the method without dividing into communities requires less time compared to the division into communities. However, after reaching a certain number of community divisions (i.e., *k* value 18 for Film Trust and Wikilens datasets), the evaluation time becomes less than that at the time taken without community division and remains constant. Similarly, in the case of the Cell Phone Recommendation dataset, the evaluation time becomes less than that at *c* value 1, and starting from *c* value 7 constant time is maintained for evaluation.

**Figure 11:**
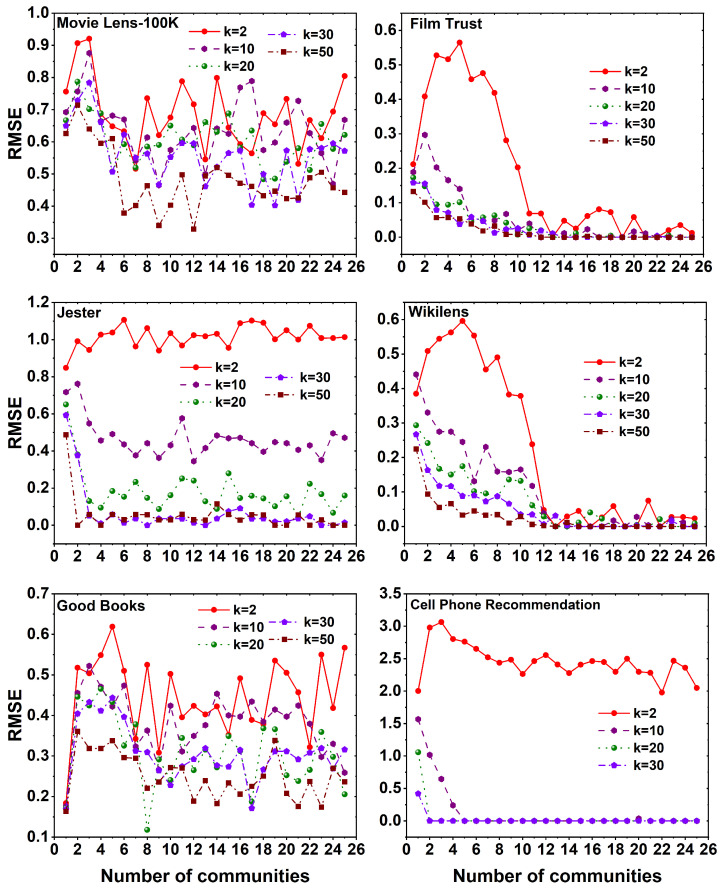
RMSE plots using FANMF method for six different datasets, namely MovieLens 100K, Film Trust, Jester, Wikilens, Good Books, and Cell Phone Recommendation with 25 communities.

The obtained RMSE values from applying the FANMF method to six distinct datasets, using different values of *k* across a set of 25 communities, are illustrated in Figure 11. In all datasets, it is evident that increasing the *k* value results in a decrease in the RMSE, regardless of the specific communities. Significantly, in the Film Trust, Wikilens, and Cell Phone Recommendation datasets, it is notable that the RMSE values stabilize after a certain number of communities. This stability is observed across all *k* values, indicating a consistent RMSE value. Moreover, across all datasets, it can be observed that a higher number of latent features leads to lower RMSE values when employing the FANMF method.

**Figure 12:**
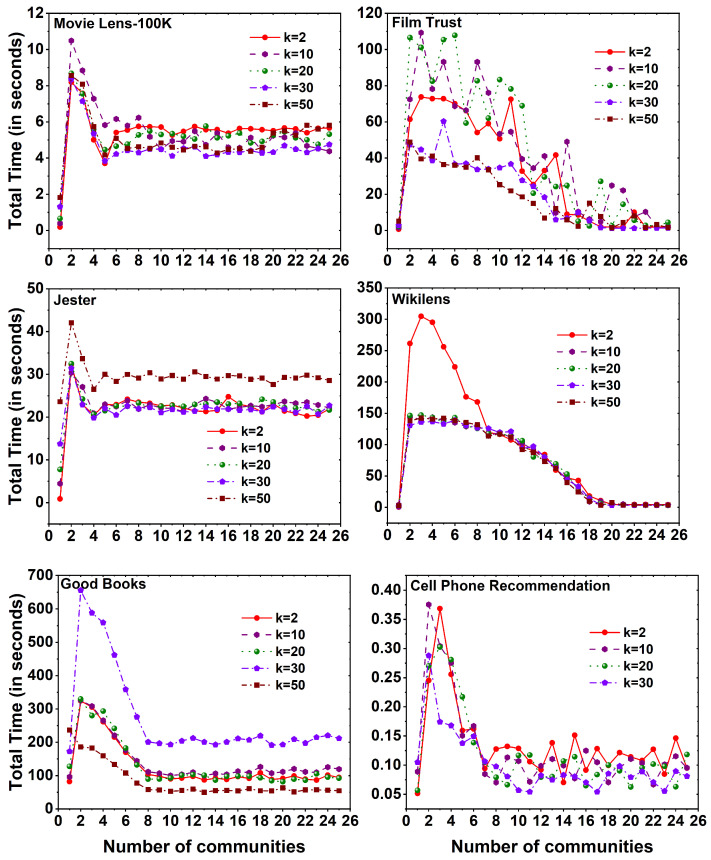
Time taken to calculate FANMF method for six different datasets, namely MovieLens 100K, Film Trust, Jester, Wikilens, Good Books, and Cell Phone Recommendation with 25 communities.

The computational time needed to run the FANMF method on six different datasets using various *k* values over a set of 25 communities is shown in Figure 12. A notable observation across all datasets is that the computation time for the community 2 exceeds that of the community 1. However, after reaching a certain number of communities, the computation time decreases compared to the community 1 for the Film Trust, Wikilens, and Good Books datasets. Conversely, for the MovieLens 100K and Jester datasets, a constant computation time is maintained, which is higher than that of the community 1.

## 7. Conclusions

In this study, we have delved into the realm of parallel computation. We proposed Community-Based Matrix Factorization (CBMF), which parallelizes matrix factorization utilizing community information. The experimental results obtained from our study provide compelling evidence of the scalability and speedup attained through CBMF, surpassing the performance of sequential implementations. These findings underscore the immense potential of parallel computation in effectively tackling the resources in the distributed environment. Looking ahead, the introduction of our parallel computation framework unlocks fresh opportunities for the efficient processing of large datasets. It empowers researchers and practitioners to extract valuable insights from intricate networks. This framework serves as a stepping stone for future advancements in providing accurate recommendations across diverse domains. It lays the foundation for future innovations and breakthroughs in the realm of data-driven solutions.

## Figures and Tables

**Table 1:** Dataset statistics for six different datasets, namely MovieLens 100K, Film Trust, Jester, Wikilens, Good Books, and Cell Phone Recommendation datasets.

Dataset	Number of Users	Number of Items	Number of Ratings	Rating Scale	Average Rating	Sparsity
**MovieLens** **100K**	943	1682	100,000	1–5	3.529	0.937
**Film Trust**	1508	2071	35,494	0.5–4	3.002	0.988
**Jester**	31,958	140	1,048,575	−10–+10	0.955	0.839
**Wikilens**	326	5111	26936	0.5–5	3.468	0.983
**Good** **Books**	13,123	7774	1,048,575	1–5	3.806	0.989
**Cell Phone** **Recommendation**	99	33	990	1–10	6.689	0.708

**Table 2:** Time taken (in seconds) to calculate the community modularity score for six different datasets, namely MovieLens 100K, Film Trust, Jester, Wikilens, Good Books, and Cell Phone Recommendation.

Dataset	Time (s)
MovieLens 100K	1473.21
Film Trust	936.00
Jester	11,714.99
Wikilens	1068.41
Good Books	43,125.11
Cell Phone Recommendation	15.58

**Table 3:** RMSE results of CBMF vs. non-CBMF.

**MF method (→)/** **Dataset (↓)**	Basic MF		SVD++		FANMF	
	**Non-CBMF**	**CBMF**	**Non-CBMF**	**CBMF**	**Non-CBMF**	**CBMF**
MovieLens 100K	3.8	0.37	1.26	0.21	0.75	0.2
Film Trust	7.69	0.002	0.4	0.003	0.2	0.0001
Jester	3.1	0.55	1.9	0.47	0.84	0.005
Wikilens	5.11	0.004	0.63	0.005	0.44	0.002
Good Books	3.9	0.11	0.55	0.02	0.18	0.009
Cell Phone Recommendation	5.8	0.42	4.7	0.7	2.9	0.003

## Data Availability

Not applicable.

## Abbreviations

Abbreviations used in this paper are as follows:

BG	Bipartite Graph
DMF	Deep Matrix Factorization
FANMF	Factorized Asymmetric Nonnegative Matrix Factorization
MF	Matrix Factorization
NLP	Natural Language Processing
NMF	nonnegative Matrix Factorization
PCSNMF	Pairwisely Constrained Nonnegative Symmetric Matrix Factorization
RMSE	Root Mean Square Error
SVD++	Advanced Singular Value Decomposition
